# Comparative effectiveness of malaria prevention measures: a systematic review and network meta-analysis

**DOI:** 10.1186/s13071-018-2783-y

**Published:** 2018-03-27

**Authors:** Kinley Wangdi, Luis Furuya-Kanamori, Justin Clark, Jan J. Barendregt, Michelle L. Gatton, Cathy Banwell, Gerard C. Kelly, Suhail A. R. Doi, Archie C. A. Clements

**Affiliations:** 1 Research School of Population Health, College of Health and Medicine, The Australian National University, ACT, Canberra, Australia; 2 Department of Population Medicine, College of Medicine, Qatar University, Doha, Qatar; 3 Centre for Research in Evidence-Based Practice (CREBP), Faculty of Health Sciences and Medicine, Bond University, Gold Coast, Queensland Australia; 4 School of Public Health, The University of Queensland, Brisbane, Queensland Australia; 5 Epigear International Pty Ltd, Sunrise Beach, Queensland Australia; 6 School of Public Health & Social Work, Queensland University of Technology, Brisbane, Queensland Australia

**Keywords:** Malaria, Preventive measures, Meta-analysis, Efficacy

## Abstract

**Background:**

Malaria causes significant morbidity and mortality worldwide. There are several preventive measures that are currently employed, including insecticide-treated nets (ITNs, including long-lasting insecticidal nets and insecticidal-treated bed nets), indoor residual spraying (IRS), prophylactic drugs (PD), and untreated nets (UN). However, it is unclear which measure is the most effective for malaria prevention. We therefore undertook a network meta-analysis to compare the efficacy of different preventive measures on incidence of malaria infection.

**Methods:**

A systematic literature review was undertaken across four medical and life sciences databases (PubMed, Cochrane Central, Embase, and Web of Science) from their inception to July 2016 to compare the effectiveness of different preventive measures on malaria incidence. Data from the included studies were analysed for the effectiveness of several measures against no intervention (NI). This was carried out using an automated generalized pairwise modeling (GPM) framework for network meta-analysis to generate mixed treatment effects against a common comparator of no intervention (NI).

**Results:**

There were 30 studies that met the inclusion criteria from 1998–2016. The GPM framework led to a final ranking of effectiveness of measures in the following order from best to worst: PD, ITN, IRS and UN, in comparison with NI. However, only ITN (RR: 0.49, 95% CI: 0.32–0.74) showed precision while other methods [PD (RR: 0.24, 95% CI: 0.004–15.43), IRS (RR: 0.55, 95% CI: 0.20–1.56) and UN (RR: 0.73, 95% CI: 0.28–1.90)] demonstrating considerable uncertainty associated with their point estimates.

**Conclusion:**

Current evidence is strong for the protective effect of ITN interventions in malaria prevention. Even though ITNs were found to be the only preventive measure with statistical support for their effectiveness, the role of other malaria control measures may be important adjuncts in the global drive to eliminate malaria.

**Electronic supplementary material:**

The online version of this article (10.1186/s13071-018-2783-y) contains supplementary material, which is available to authorized users.

## Background

Malaria imposes a great health and socio-economic burden on humanity, with an estimated 3.2 billion people at risk of being infected with malaria [1]. In 2016, there were approximately 216 million cases with 445,000 deaths, most of which were in children aged under 5 years in Africa [1]. Between 2000 and 2015, it has been estimated that there was a 37% global reduction in malaria incidence [2]. This improvement was likely made possible by economic development and urbanization in many endemic countries [3] as well as a substantial increase in investment in tackling malaria [4], leading to an increase in preventative activities, and improved diagnostics and treatment. The Global Technical Strategy for Malaria 2016–2030 (GTS) has a target to eliminate malaria in at least ten countries by 2020, 20 countries by 2025, and 30 countries by 2030 [2, 5].

Vector control remains an essential component of malaria control and elimination. The capacity of vectors to transmit parasites and their vulnerability to vector control measures vary by mosquito species and are influenced by local environmental factors. Personal preventive measures that prevent contact between the adult mosquitoes and human beings are the main methods of prevention currently in practice. These include insecticide-treated nets (ITNs), and indoor residual spraying (IRS) [6]. ITNs are of two types: long-lasting insecticidal nets (LLINs) that have the insecticide incorporated into fibers during the manufacturing process, which leads to a longer duration of effectiveness and insecticide-treated nets (ITNs) which are impregnated with insecticides every six months. Indoor residual spraying (IRS) involves spraying insecticides on the walls of the houses. Additionally, antimalarial chemoprophylaxis is used for prevention of malaria in children and pregnant women. The commonly used prophylactic drugs (PD) are sulphadoxine-pyrimethamine (SP), mefloquine (MQ), amodiaquine (AQ), dihydroartemisinin-peperaquine (DP) and artesunate (AS). The main advantage of using PD is that they only require a single dose to achieve a full prophylactic effect [7, 8]. However, the most common PD is SP and it is becoming less effective due to resistance [9–13]. As a result, other drugs such as MQ and AQ are increasingly being used as a substitute for or in combination with SP [12, 14]. MQ provides a longer period of prophylaxis but side effects (agranulocytosis in 1 per 2000 patients) [15] are the main problem [16, 17]. Similarly, AQ has been used in combination with SP but AQ is not well tolerated [14]. Many other less commonly utilized measures include insecticide-treated curtains (ITC), mosquito coils, insecticide-treated hammocks, and insecticide-treated tarpaulins.

There has been a decrease in malaria incidence worldwide, but what remains unclear is which of the common preventive interventions is the most effective for prevention of malaria infection. This knowledge may help prioritise resourcing of these interventions. There has been one comparative study of preventive efficacy that compares mortality across ITN, IRS and PD and this study demonstrated that the impact of IRS is equal to that of ITN on reducing malaria-attributable mortality in children [18]. There have also been several systematic reviews and meta-analyses focusing on single preventive measures. These reviews of existing data suggest that PD [19–22], is effective in preventing malaria infection in children when treated on a monthly basis with no protection when given three-monthly. The reviews of both ITN [23–25] and IRS [26, 27] provide support for their effectiveness as malaria preventive measures, but there is no data on the effectiveness of one measure over another.

Therefore, this study aims to present an up-to-date comparison of the effectiveness of the four common malaria preventive measures (ITNs, UNs, IRS and PDs) for which data are readily available and compare these against no intervention [NI, defined as no intervention or placebo or a study group with standard care (any intervention given to all participants)]. A network meta-analysis methodology was chosen to pool the data as it allows comparisons of multiple preventive measures simultaneously and allows comparisons across preventive measures not directly tested in the included trials (indirect comparisons across a pair of studies that share a common comparator). In addition, this method allows ranking of the effectiveness of these measures for decision making.

## Methods

### Search strategy and eligibility criteria

A systematic literature review was undertaken using four medical and life sciences databases (PubMed, Cochrane Central, Embase and Web of Science). They were searched from their inception to March 2016 for trials that compared the effectiveness of malaria preventive measures. Search terms included were “*malaria*”, “*Plasmodium falciparum*”, “*Plasmodium vivax*”, “*bed net*”, “*mosquito control*”, “*antimalarial*”, and *“insecticides”*; the specific keywords and connectors for each database are listed in the Additional file 1: Table S1.

The inclusion of studies were restricted to (i) interventional studies; (ii) conducted in humans (with no restriction of age or sex); (iii) that compared two or more of the following malaria preventive measures: ITN, UN, IRS, PD or NI; and (iv) reported the number of new malaria cases diagnosed through microscopy or rapid diagnostic tests (RDT) after each intervention compared amongst a population at risk over time. Exclusion criteria included: (i) non-intervention studies; (ii) conference abstracts; and (iii) other less commonly utilized malaria preventive measures including ITC, mosquito coils, insecticide-treated hammocks and insecticide-treated tarpaulins. No language restrictions were imposed. Since we used a generalized pairwise modeling approach (see below), odd numbers of treatments (e.g. three treatment arms) required selection of a pair for inclusion in this study and we therefore excluded the arm that had the most available data in this synthesis [(i) arms that we excluded do not make a difference, (ii) concurrent interventions and no effect modification].

### Study selection and data extraction

The citation search was developed and executed by JC, followed by selection of citations by title and abstract independently by two researchers (KW and LFK). The selected studies underwent a full-text review for all potentially relevant studies. Data from the included studies were then independently extracted in a spreadsheet by the same two researchers. The extracted data included: (i) the country of study; (ii) year(s) when the study was conducted; (iii) study design; (iv) study population characteristics; (v) preventive measures employed in the trial; and (vi) the number of new cases of malaria and person-months at risk. The extracted data were then cross-checked by the two researchers and any discrepancies during the selection of studies or data extraction were resolved through discussion and consensus following independent evaluation by another author (SARD).

### Statistical analysis

The outcome of interest was the rate ratio (RR) of new malaria cases in intervention-A *vs* intervention-B following the implementation of different preventative measures. An automated generalized pairwise modeling (GPM) framework [28] was used to generate mixed treatment effects against a common comparator (NI). This framework is an extension of the Bucher method [29] that automates the single three-treatment loop method. This analysis starts by pooling effect sizes based on direct comparisons between any two interventions using meta-analytic methods. The indirect comparison was then performed by automated generation of all possible closed loops of three-treatments such that one of them was common to the two studies and formed the node where the loop began and ended but where the common node was never NI, while one of the other nodes was always NI. Finally, the mixed effects (multiple direct/indirect effects) were pooled using the same meta-analysis model as used for pooling direct effects. The analysis therefore led to a final mixed treatment effect estimate for different interventions versus NI. Estimates of preventive effectiveness were then ranked by their point estimates. It should be pointed out that it is common for network plots based on Bayesian methods to rank treatments by the surface under the cumulative ranking curve (SUCRA). From our frequentist perspective, treatment effects are thought of as fixed parameters and thus, strictly speaking SUCRA does not apply. A frequentist alternative called the P-score has been proposed but SUCRA or P-scores have no major advantage compared to what we have done, i.e. ranking treatments by their point estimates [30].

All direct estimates were pooled using the inverse variance heterogeneity (IVhet) model [31] as were all mixed estimates, but this synthesis process was also repeated using the random effects model for comparison (the random effects analysis was undertaken under the GPM framework as well as under the frequentist multivariate meta-analysis framework for comparison (see Additional file 1: Tables S2 and S3 for details).

Cluster randomized controlled trials (RCT) were combined with other study types after accounting for clustering using the design effect (DEFF). The DEFF was calculated as follows:

$$ DEFF=1+\rho \left(c-1\right) $$

where *ρ* is the intra-class correlation for the statistic in question and *c* is the average size of the cluster. We then divided the numbers in each 2 × 2 table of the study by the DEFF to calculate a corrected sample size, which was then utilized in the meta-analysis. Different units of clusters such as villages and households were used in different studies. The intra-class correlation coefficient (*ρ*) was provided only in one study [32], and this (*ρ* = 0.048) was used for calculation of the DEFF for other cluster RCT studies.

Statistical heterogeneity across direct effects pooled in the meta-analysis were assessed by the Cochran’s *Q* and the *H* index which is the square root of *H*^2^, the estimated residual variance from the regression of the standardized treatment effect estimates against the inverse standard error in each direct meta-analysis. *H* was computed as follows:

$$ H=\sqrt{\frac{\max\ \left[\max \left(1,n-1\right),Q\right]}{\max \left(1,n-1\right)}} $$

where *n* is the number of study estimates pooled and Q represents the Chi squared from Cochran’s *Q*.

Transitivity was assessed statistically by looking at inconsistency across the network as a whole using the weighted pooled *H* index ($\overline{H}$) which was computed as follows from the Cochran’s *Q* statistic for the *k* final comparisons:

$$ \overline{H}=\sqrt{\frac{\sum_{j=1}^k\max\ \left[\max \left(1,n-1\right),Q\right]}{\left({\sum}_{j=1}^kn\right)-k+s}} $$

where *n* is the number of estimates pooled across each comparison and *s* is the number comparisons (out of *k*) were *n* = 1. The minimum value *H* or $\overline{H}$ can take is 1, it is not influenced by *n*, and $\overline{H}<3$was taken to be minimal inconsistency based on our simulations of *H* in homogenous direct meta-analyses [28].

Sensitivity analyses were undertaken through limiting the network to (i) studies conducted in children or (ii) studies including only *Plasmodium falciparum* infection and then re-running the GPM analysis.

Publication bias was assessed using a ‘comparison adjusted’ funnel plot where on the horizontal axis the difference of each study’s observed ln(RR) from the comparison’s mean ln(RR) obtained from the pairwise fixed effect meta-analysis was plotted. In the absence of small-study effects, we expect the studies to form an inverted funnel centred at zero [33]. All the analyses involved in the generalised pairwise modelling (GPM) framework for multiple indirect and mixed effects were conducted using MetaXL v5.2 (EpiGear International, Sunrise Beach, Australia) [28]. Funnel and network plots were produced using Stata version 13 (Stata Corporation, College Station, TX, USA).

### Quality assessment

The quality of the included studies was assessed using a modification of a quality checklist used in another study by one of the authors [34]. The studies were assessed on inclusion of safeguards relating to study design, selection, information, blinding of study assessors, and analytical biases. There were 12 questions with a possible maximum count of 17 safe-guards (Additional file 1: Table S4).

## Results

### Data extraction

**Fig. 1:**
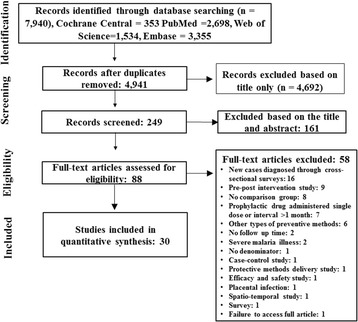
Search flowchart. *Note*: details of excluded studies in Additional file 1: Table S5

The search strategy identified 7940 citations (Cochrane Central = 353, PubMed = 2698, Web of Science = 1534 and Embase = 3355). After deleting duplicate citations, a total of 4941 citations were retrieved for the initial screening. Of these, 4692 citations were excluded based on title only. Records of 249 citations were screened and 161 citations were excluded based on the title and abstract. Eighty eight articles were assessed for eligibility, of which 58 articles were excluded (Additional file 1: Table S5). Thirty citations fulfilled eligibility criteria and were included in the meta-analysis (Fig. 1). Data from the included studies were extracted and summarized in a spreadsheet (Table 1).

**Table 1:** Characteristics of included studies

Reference	Study area, year of trial	Study design	Study population	Diagnosis	I1	Malaria incidence/person months	I2	Malaria incidence/ person months	I3	Malaria incidence/ person months	I4	Malaria incidence/ person months
Beach et al., 1993 [35]	Kenya, 1990	Quasi-experimental- with control group	Children < 6 years in 6 villages	Microscopy	ITN^b^	82/195	NI^b^	136/193	ITC	170/224	–	–
Charlwood et al., 2001 [36]	Eastern Sudan, 1997	Cluster RCT	All ages	PCR and microscopy	IRS	46/418	NI	38/451	–	–	–	–
Cisse et al., 2006 [37]	Sahel and sub-Sahel region, 2002–03	RCT	Children 2–59 months	Microscopy	PD	39/1315	NI	222/1321	–	–	–	–
Dicko et al., 2011 [38]	Mali, 2008	RCT	Children 3–59 months	Microscopy	PD	149/4346	NI	832/4148	–	–	–	–
Fraser-Hurt, 1999 [39]	Tanzania, 1996	RCT	Children 5–24 months	Microscopy	ITN	173/360	NI	234/360	–	–	–	–
Hale, 2003 [40]	Ghana, 1998	RCT	Adults	Microscopy	PD	6/131	NI	86/117	–	–	–	–
Hamel, 2011 [41]	Kenya, 2000	Quasi-experimental-control group	All ages > 6 months	RDT	IRS+ITN	114/7424	ITN	251/6840	–	–	–	–
Hill et al., 2014 [62]	Yunnan, China, 2007	RCT	All ages	Microscopy and RDT	ITN	12/10,854	NI	73/10,857	–	–	–	–
Kamol-Ratanakul et al., 1992 [53]	Thailand, 1987–88	RCT	Adults	Microscopy	ITN	28/1026	UN	51/1099	–	–	–	–
Konate et al., 2011 [42]	Ghana, 2004	RCT	Children < 6 years	Microscopy	ITN	332/4564	NI	982/3504	–	–	–	–
Kroeger et al., 1995 [63]	South America, 1991–94	Quasi-experimental-control group	All ages	Microscopy and RDT	ITN	899/38,260	NI	1617/40,668	–	–	–	–
Kweku et al., 2008 [43]	Ghana, 2005–06	RCT	Children 3–59 months	Microscopy	PD (monthly)^b^	44/3756	PD (bimonthly)	112/3678	PD (monthly)	109/3372	NI^b^	183/3900
Luxemburger et al., 1994 [54]	Thailand-Burma, 1990–91	RCT	School children 4–15 years	Microscopy	ITN	29/933	UN	52/939	–	–	–	–
Lwin et al., 2012 [59]	Thailand, 2006–08	RCT	Adults	Microscopy	PD	5/2000	NI	69/845	–	–	–	–
Marbiah et al., 1998 [44]	Sierra Leone, 1992–93	Cluster RCT	Children 3 months to 6 years	Microscopy	ITN	48/590	NI	90/559	–	–	–	–
Mwangi et al., 2003 [45]	Kenya, 2000	Quasi-experimental-control group	Children < 19 years	Microscopy	UN^b^	62/638	UN^a^	22/42	NI^b^	58/383	–	–
Nankabirwa et al., 2014 [46]	Uganda, 2011–12	RCT	Children 6–14 years	Microscopy	PD (monthly)^b^	3/2886	PD (school term)	81/2857	NI^b^	83/2912	–	–
Nevill et al., 1988 [47]	Kenya, NR	RCT	Children 6–18 years	Microscopy	UN^b^	1/130	PD^b^	8/123	NI	35/123	–	–
Odhiambo et al., 2010 [48]	Kenya, 2004–08	RCT	Infants 5–16 weeks	Microscopy	PD^e^	2/334	PD	2/334	NI^e^	17/325	–	–
Pinder et al., 2015 [49]	Gambia, 2010–11	Cluster RCT	Children 6 months to 14 years	Microscopy and RDT	IRS	15/886	NI	17/899	–	–	–	–
Rowland et al., 1996 [55]	Pakistan, 1991	Quasi-experimental- control group	All ages	Microscopy	ITN	235/8388	NI	541/8364	–	–	–	–
Sahu et al., 2003 [57]	India, 1999	Cluster RCT	All ages	Microscopy	ITN	3/551	NI	9/565	–	–	–	–
Sesay et al., 2011 [50]	Gambia, 2008	RCT	Children < 5 years	Microscopy	PD	1/2248	NI	3/2279	–	–	–	–
Sexton et al., 1990 [51]	Kenya, 1988	RCT	All ages	Microscopy	ITN^e^	55/339	ITC	34/323	NI^e^	75/322	–	–
Shah et al., 2013 [61]	India, 2006–11	Quasi-experimental- control group	All ages	Microscopy	ITN	124/34,272	NI	45/18,624	–	–	–	–
Sharma et al., 2009 [58]	India, 2006–07	Cluster RCT	All ages	Microscopy	ITN^e^	16/23,436	UN^b^	48/24,228	NI	50/12696	–	–
Smithuis et al., 2013 [32]	Myanmar, 1998	Cluster RCT	Children < 10 years	Microscopy	ITN	351/3989	NI	713/4053	–	–	–	–
Snow et al., 1988 [52]	Gambia, 1986	Cluster RCT	Children 1–9 years	Microscopy	UN	22/435	NI	20/303	–	–	–	–
Soleimani-Ahmad et al., 2012 [60]	Iran, 2009–10	Cluster RCT	All ages	Microscopy	ITN	17/7008	NI	35/7145	–	–	–	–
Taylor et al., 1999 [56]	Indonesia, 1996-97	RCT	Adult (non-pregnant) 18–55 years	Microscopy	PD^b^	28/467	PD	3/265	NI^e^	56/142	–	–

*Abbreviations*: *I1* intervention 1, *I2* intervention 2, *I3* intervention 3, *I4* intervention 4, *MI* Malaria infection, *ITC* insecticide-treated curtain, *ITN* insecticide-treated net, *PD* prophylactic drug, *NI* no intervention, *RDT* rapid diagnostic test, *RCT* randomized clinical trial, *IRS* indoor residual spraying, *UN* untreated nets

^a^Untreated net torn

^b^Included arms in network meta-analysis if more than two arms

### Characteristics of included studies

The literature search on malaria control and preventive measures led to the identification of the five treatment groups across the studies (ITN, UN, PD, IRS and NI). A total of 30 studies were included in the current meta-analysis. These studies were conducted from 1988 to 2015. Eighteen studies were conducted in Africa [35–52], 11 studies were from Asia [32, 53–62] and one study from South America [63]. Ten studies did not restrict study participants to any age [36, 41, 51, 55, 57, 58, 60–63], four studies only included adults as study participants [40, 53, 56, 59], and the rest of the studies (16) were conducted in children and adolescents (0–19 years) [32, 35, 37–39, 42–50, 52, 54]. There were 21 studies that reported *P. falciparum* infection rates separately [32, 35, 37–43, 45–48, 50–52, 54, 55, 58, 60, 62]. The latter two groups were used in a sensitivity analysis (see below). The most common study design was the RCT with 16 studies [37–40, 42, 43, 46–48, 50, 51, 53, 54, 56, 59, 62], eight studies were cluster RCT [32, 36, 44, 49, 52, 57, 58, 60], and the rest (6) were quasi-experimental studies with a control group [35, 41, 45, 55, 61, 63]. Twenty one studies had two arms [32, 36–42, 44, 49, 50, 52–55, 57, 59–63], eight studies had three arms, [35, 45–48, 51, 56, 58] and one had four arms [43]. Of those with three arms we dropped the curtain arm in two studies [31, 51] (not part of this review) and one of the PD arms in four other studies [43, 46, 48, 56] that reported PD comparisons at different dosages or intervals. Microscopy was used for detection of *Plasmodium* parasites in 25 studies [32, 35, 37–40, 42–48, 50–61], three studies used both microscopy and RDTs [49, 62, 63], and one study each used RDT [41] and polymerase chain reaction (PCR) and microscopy [36] for diagnosis (Table 1).

### Interventions utilized across studies

Twenty five studies had a NI arm [32, 35–46, 48–52, 55–57, 59, 61–63], and seven studies had a UN arm [45, 47, 52–54, 58, 60]. Of the eleven studies that used a PD arms [37, 38, 40, 42, 43, 46–48, 50, 56, 59], the regimens were all different (Additional file 1: Table S6). Fourteen studies reported the use of ITN in one arm [32, 35, 39, 44, 51, 53–55, 57, 58, 60–63]. In these studies, different types of nets and insecticides for treating such nets were used (Additional file 1: Table S7). The insecticides used for IRS in the three studies of this intervention were also different and are listed in (Additional file 1: Table S8) [36, 41, 49].

### Quality assessment

The quality of the studies including types of study, randomization and other characteristics was assessed through 17 safeguards against bias as outlined in the supplementary material. They were combined into a univariate overall quality score consisting of counts of safeguards ranging between 7 and 17 out of a maximum possible of 17. The ranges of the scores were 10–17, 7–14, 9–16, 10–16, and 8–11 in PD *vs* NI, ITN *vs* NI, IRS *vs* NI, ITN *vs* UN, and UN *vs* NI studies, respectively. The most common safeguards missing were consideration of confounders such as socio-economic status, owning LLINs, malaria prevalence and blinding of assessors in between 46.7–93.3% of studies (Additional file 1: Table S9).

### Quantitative synthesis

**Fig. 2:**
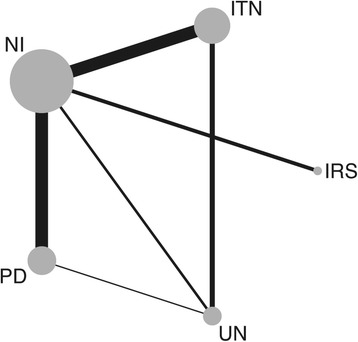
Network plot showing the comparison groups. The circle size is proportional to the number of studies including that intervention while line width is proportional to the number of comparisons. *Abbreviations*: ITN, insecticide-treated nets; UN, untreated net; IRS, indoor residual spraying; NI, no intervention; PD, prophylactic drug

Seven direct estimates based on head-to-head comparison within 30 studies, which included 60 treatment groups, were available (Table 2 and Fig. 2). In these direct comparisons, PD (RR: 0.21, 95% confidence interval [CI] 0.13–0.33), ITN (RR: 0.57, 95% CI: 0.41–0.81), and UN (RR: 0.67, 95% CI: 0.49–0.92) were significantly better than NI. Similarly, UN (0.12, 95% CI: 0.01–0.94) was better as compared to PD, and IRS (RR: 0.55, 95% CI: 0.20–1.56) was not significantly different from NI.

**Table 2:** Direct, indirect and final results from comparison of different preventive measures

ID	Comparison	Active	Control	RR	95% LCI	95% HCI	*K* ^a^	*H*
Direct estimates								
1	UN-PD	UN	PD	0.12	0.01	0.94	1	1
2	ITN-UN	ITN	UN	0.56	0.40	0.76	4	1.11
3	ITN-NI	ITN	NI	0.57	0.41	0.81	10	2.76
4	PD-NI	PD	NI	0.21	0.13	0.33	10	2.49
5	IRS-NI	IRS	NI	0.55	0.20	1.56	3	3.41
6	UN-NI	UN	NI	0.67	0.49	0.92	2	1
Indirect estimates (source IDs)								
7	Indirect UN *vs* NI (1, 4)	UN	NI	0.02	0.003	0.21	2	3.01
8	Indirect ITN *vs* NI (2, 6)	ITN	NI	0.37	0.24	0.58	2	1.85
9	Indirect PD *vs* NI (1, 6)	PD	NI	5.70	0.70	46.58	2	3.01
10	Indirect UN *vs* NI (2, 3)	UN	NI	1.03	0.64	1.66	2	1.85
Final estimates from all evidence (source IDs)								
	PD (4, 9)	PD	NI	0.24	0.004	15.43	2	3.00
	ITN (3, 8)	ITN	NI	0.49	0.32	0.74	2	1.85
	IRS (5)	IRS	NI	0.55	0.20	1.56	1	1
	UN (6, 7, 10)	UN	NI	0.73	0.28	1.90	3	2.59
Network H = 2.21								

*Abbreviations*: *ITC* insecticide-treated curtain, *ITN* insecticide-treated net, *PD* prophylactic drug, *NI* no intervention, *IRS* indoor residual spraying, *UN* untreated nets, *RR* rate ratio, *LCI* lower confidence interval, *HCI* higher confidence interval

^a^Number of studies

The indirect estimate for ITN (RR: 0.37, 95% CI: 0.24–0.58) was consistent with the direct estimate, while that for PD (RR: 5.70, 95% CI: 0.70–46.58) demonstrated an inconsistent and very uncertain effect as opposed to the direct estimate. UN had two indirect estimates possible and both were inconsistent with the direct effect but in opposite directions with either a grossly positive effect (RR: 0.02, 95% CI: 0.003–0.21) or a negative effect (RR: 1.03, 95% CI: 0.64–1.66) (Table 2).

**Fig. 3:**
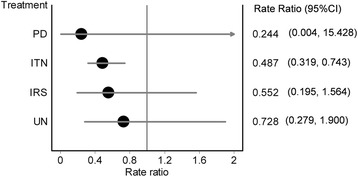
Results of network meta-analysis of 30 studies comparing listed interventions against NI. Only the PD-NI mixed effects showed modest inconsistency and this is reflected in the marked uncertainty (wide 95% confidence intervals) of the effect estimate

The final estimates were based on all evidence for these interventions in comparison with NI and results showed that, PD (RR: 0.24, 95% CI: 0.004–15.43), ITN (RR: 0.49, 95% CI: 0.32–0.74), IRS (RR: 0.55, 95% CI: 0.20–1.56), and UN (RR: 0.73, 95% CI: 0.28–1.90) were all less likely to be associated with incident infection as compared to participants using no preventive measure (NI). However, only ITN demonstrated a statistically significant effect (Table 2 and Fig. 3).

There was overall minimal statistical network inconsistency ($\overline{H}=2.21\Big)$over comparisons despite the inconsistent direct and indirect effects, because of the huge uncertainty associated with indirect effects possibly reflecting heterogeneity in terms of the geographical locations and population characteristics of studies. One final effect (PD-NI) demonstrated modest inconsistency (*H* = 3.0) while the rest demonstrated minimal to no inconsistency (H < 3, see Table 2), again because of uncertainty around the individual mixed effects.

### Sensitivity analysis and publication bias

Heterogeneity was evident when selection criteria were modified to include only children or only *P. falciparum* infections respectively (with $\overline{H}$ at 2.35 and 2.29, respectively (Additional file 2: Figure S1; Additional file 3: Figure S2). However, the rank of effectiveness of different preventive measures remained unchanged in both analyses except that ITN was less effective than IRS in only *P. falciparum* and effects were now less precise because numbers of studies were lower.

The comparison-adjusted funnel plot demonstrated little evidence of asymmetry except for the PD-NI comparison, which was in keeping with the fact that there was both considerable heterogeneity and inconsistency across this comparison (Additional file 4: Figure S3).

## Discussion

This meta-analysis showed that only ITNs had a significant effect in protection against malaria infection. While the effect size for PD was larger, the uncertainty was high, thus making the impact of this intervention uncertain. These findings confirm that impregnated insecticides on ITNs offers better protection than UNs in preventing mosquitoes from taking a blood meal from the host through its excito-repellency effect [23, 64–70]. The insecticides on ITNs may also inhibit mosquitoes from entering a house similar to the effect of IRS. Mortality of mosquitoes in the range of 25–75% has been observed after they enter huts in search of blood meals irrespective of the various different pyrethroids used in ITNs [67]. Individual studies on efficacy of this intervention have shown that the risk of malaria infection due to ITN use can reduce by up to 39–62% and child mortality by 14–29% [24, 71]. Interestingly, the impact of ITNs on child mortality and morbidity have been reported to extend out from areas with the actual ITN use to neighbouring areas because of the impact of the insecticidal nets on the entomological inoculation rate (EIR) of the local vector population [72–74]. Similarly, mathematical modelling has shown that ITNs can even protect against mosquitoes that feed outdoors [75]. ITNs have also been reported to protect women in pregnancy and in reducing placental malaria, anaemia, stillbirths and abortions [65]. Of note, the combination of IRS and ITN has been shown to offer better protection as compared to ITNs alone [27, 41, 76–79]. Only one of the latter studies was included in our synthesis which compared IRS *vs* NI where both arms were also given ITNs and the RR was 0.42 (95% CI: 0.34–0.52) suggesting that the effects are independent and additive on malaria prevention [41]. The other studies did not meet our inclusion criteria because these studies were cross-sectional and pre-post interventional studies but evidence from them was also supportive of this conclusion.

Despite reports of pyrethroid resistance in parts of the world including Africa [80–85], ITNs treated with pyrethroids continue to provide significant protection against malaria [69, 71, 86, 87]. ITNs of the LLIN type have insecticides impregnated in the fibres of nets, which are wash resistant for the four- to five-year lifespan of the ITNs. ITBN types of ITNs require insecticides to be impregnated every six months. Due to reduced costs and ease of implementation, the LLINs have gained huge popularity in recent years and given their superiority to IRS in this analysis as well as in previous studies [27, 88], this would represent a strong choice in terms of malaria prevention.

The biggest effect size was for PD. This intervention prevents or reduces the incidence of malaria primarily through clearing existing parasitaemia (or reducing it to a level below the fever threshold) and preventing new infections [8, 89, 90]. In our analysis however, we found the least precision for the effect estimate and the most inconsistency, suggesting that the effects varied widely across studies. Whilst the effectiveness of prophylactic drugs has been documented in children and pregnant women in sub-Saharan Africa, it has not been substantiated in other parts of the world [38, 42, 91, 92], possibly because of the limited ability of drugs to prevent relapse in *P. vivax* infection [8, 93, 94]. Nevertheless, in our analysis restricted to *P*. *falciparum,* the same uncertainty was observed for PDs as in the full dataset. There are other concerns apart from preventive efficacy with the use of drugs as they can also result in impairment of natural immunity, and rebound infections of the children who received chemo-prophylaxis for 1–5 years [95–98]. The widespread use of chemoprophylaxis in children and pregnant women could possibly increase the rate of spread of drug resistance [99].

The preventive measure with the next highest effect estimate was IRS, a critical component of the WHO’s Global Malaria Eradication Program from 1955–1969 and the main intervention attributed to the elimination or dramatic reduction of malaria in parts of Europe, Asia and Latin America [27]. The basic principle of IRS in vector control is that IRS protects inhabitants against mosquito bites by killing the blood-fed females who rest on the walls after feeding and also protect inhabitants against mosquito bites by diverting the vector from entering a sprayed house an effect known as excito-repellency [100, 101]. If the mosquito does enter the house, after biting, the female mosquito eventually rests on sprayed surfaces, where it picks up a lethal dose of insecticide, thus preventing transmission of the parasite to others. In a village with a high percentage coverage of houses with IRS, the mean age of the village mosquito population is expected to be reduced and very few mosquitoes will survive the approximately 12 days required for sporozoite maturation to be able to transmit the parasites [71]. Thus, IRS reduces malaria transmission at the community level by reducing mosquito longevity and abundance, but it has also been reported to provide household-level protection [27]. Studies have shown that IRS was more effective with high initial prevalence, multiple rounds of spraying and in regions with a combination of *P. falciparum* and *P. vivax* [26]. Despite all the advantages of IRS, our analysis suggested a consistently better (or at worst equivalent) efficacy for ITNs compared to IRS. Mosquito mortality has been shown to decline after the third month following IRS and by the fifth month, effectiveness reduces by 12% [102]. Efficacy might wane if walls are replastered or painted following implantation of IRS, and mosquito resistance to insecticides can emerge. In addition, there is a need for trained personnel for application of insecticides, which means IRS might not always be done effectively.

Untreated nets were the least effective in preventing malaria infection as compared to other preventive measures. UNs can offer a barrier against the bite of mosquitoes; however, mosquitoes can rest on the UNs while seeking opportunities to feed on the hosts sleeping under the nets, which can be presented when any part of a host’s body comes in contact with the nets. This happens often when hosts are in a deep sleep, especially under inadequately spaced or small nets. Untreated nets can even offer resting places to mosquitoes in an IRS-sprayed house and thus cannot be recommended given the other alternatives that exist. Finally, torn untreated nets have been shown to offer no additional protection as compared to not using nets [45, 103].

A key strength of this analysis is the use of the GPM framework which avoids approximations and assumptions that are not stated explicitly or verified when the method is applied. On the contrary, the multivariate frequentist framework assumes that if there is no common comparator in the network, this then has to be handled by augmenting the dataset with fictional arms with high variance. This is not very objective and requires a decision as to what constitutes a sufficiently high variance [104]. Another alternative, the Bayesian framework, also has its problems such as requiring prior distributions to be specified for a number of unknown parameters and choices regarding over-dispersed starting values for a number of independent chains so that convergence can be assessed. While we have several choices for the meta-analytic framework, this choice may be less important than other choices regarding the modelling of effects [105]. Indeed, we were able to use the inverse variance heterogeneity model for direct estimates which has correct error estimation when compared with the random effects model [31]. Results from a random effects model (using both the multivariate meta-analysis framework as well as the GPM framework) differ slightly from our main results, especially regarding PD, which has spuriously precise estimates using this approach (Additional file 1: Tables S2, S3).

There are limitations of this study worth noting. Even though clinical and statistical significance was found for ITNs, in reality the effectiveness of interventions (ITN) are dependent on a number of extrinsic factors such as population behaviour and vector aetiology. Studies have shown that ITN use is influenced by social behaviour including education, level of knowledge on malaria, and ease of use [106, 107]. In addition, other socio-economic factors such as working and staying overnight in the forest decreases protection despite high proportion of coverage by ITNs [108–110]. Secondly, different insecticides being used for IRS and ITN over the study period would have impacted the findings of this study and the development of insecticide resistance would undermine the effectiveness of ITNs in preventing malaria. Thirdly, the methods of diagnosis of incident malaria were different in the studies. Since most of these studies were conducted in intense malaria transmission areas, this effect is however likely to be minimal. Fourthly, the vectors were different depending on the region of the study; for instance, the commonest malaria vectors in the Asian region including *Anopheles dirus*, *An. baimaii* and *An. minimus* [111, 112], are able to avoid indoor sprayed surfaces because of their exophilic and exophagic characteristics [113–115] rendering most domicile-based interventions, like ITNs and IRS less effective [114, 116]. Of the three main vectors in the African region: *An. arabiensis*, *An. funestus* and *An. gambiae* [113, 117], only *An. arabiensis* shows feeding preferences for both indoors and outdoors while the other two are indoor-feeders [117]. Other challenges include insecticide resistance [118]. Finally, the drug types and regimens varied between studies. All of these limitations have the potential to increase heterogeneity between the included studies and make it more difficult to estimate the effects of the different interventions more precisely than what we have reported.

## Conclusions

Even though ITNs were found to be the only preventive measure with statistical support for its effectiveness in this study, the role of all malaria control measures are important in the global drive to eliminate malaria. However, when a choice needs to be made for resource allocation, the results reported here tend to favour the use of ITNs.

## Additional files

Additional file 1: **Table S1.** Search Strategy. **Table S2.** Meta-analysis of different control measures against NI using the random effects model under the generalized pairwise modeling (GPM) framework in MetaXL. **Table S3.** Meta-analysis of different control measures against NI using the random effects model under the frequentist multivariate meta-analysis framework (mvmeta) in Stata. **Table S4.** Quality scale. **Table S5.** Summary of the excluded studies. **Table S6.** Drugs used in the included studies. **Table S7.** Description of ITN’s used across the studies. **Table S8.** Description of IRS treatments used across the included studies. **Table S9.** Quality assessment scores of included studies. (DOCX 56 kb)

Additional file 2: **Figure S1.** Results of network meta-analysis of 21 studies with children as a study population. (TIFF 624 kb)

Additional file 3: **Figure S2.** Results of network meta-analysis of 28 studies with incidence of *Plasmodium falciparum*. (TIFF 631 kb)

Additional file 4: **Figure S3.** Funnel plot depicting asymmetry for the PD-NI comparison. (TIFF 1482 kb)

## Abbreviations

AQ: Amodiaquine
AS: Artesunate
CI: Confidence interval
DEFF: Design effect
DP: Dihydroartemisinin-peperaquine
EIR: Entomological inoculation rate
GPM: Generalized pairwise modelling
GTS: Global Technical Strategy for Malaria 2016–2030
HCI: Higher confidence interval
IRS: Indoor residual spraying
ITC: Insecticide-treated curtain
ITN: Insecticide-treated net
IVhet: Inverse variance heterogeneity
LCI: Lower confidence interval
LLIN: Long-lasting insecticidal nets
MI: Malaria infection
MQ: Mefloquine
NI: No intervention
PD: Prophylactic drugs
RCT: Randomized controlled trials
RDT: Rapid diagnostic tests
RR: Rate ratio
SP: Sulphadoxine-pyrimethamine
SUCRA: Surface under the cumulative ranking curve
UN: Untreated nets
WHO: World Health Organization
