# Supplementary material for: Comparative effectiveness of malaria prevention measures: a systematic review and network meta-analysis
Source: Parasit Vectors. 2018 Mar 27;11:210. doi: 10.1186/s13071-018-2783-y (PMC5869791; doi:10.1186/s13071-018-2783-y)
Supplement: Supplementary file 1 — Table S1. Search Strategy. Table S2. Meta-analysis of different control measures against NI using the random effects model under the generalized pairwise modeling (GPM) framework in MetaXL. Table S3. Meta-analysis of different control measures against NI using the random effects model under the frequentist multivariate meta-analysis framework (mvmeta) in Stata. Table S4. Quality scale. Table S5. Summary of the excluded studies. Table S6. Drugs used in the included studies. Table S7. Description of ITN’s used across the studies. Table S8. Description of IRS treatments used across the included studies. Table S9. Quality assessment scores of included studies. (DOCX 56 kb) [file 13071_2018_2783_MOESM1_ESM.docx]

**Additional file**

**Table S1. Search strategy**

| **Pubmed**  "Malaria"[Mesh] OR "Plasmodium falciparum"[Mesh] OR "Parasitemia"[Mesh] OR Malaria[tiab] OR Plasmodium[tiab] OR Falciparum[tiab] OR Parasitemia[tiab]  AND  "Culicidae"[Mesh] OR "Insect Vectors"[Mesh] OR Mosquito[tiab] OR Mosquitos[tiab] OR Mosquitoes[tiab] OR Vector[tiab] OR Insect[tiab] OR Insects[tiab] OR Transmission[tiab] OR Infection[tiab] OR Infections[tiab] OR Infected[tiab]  AND  “Mosquito Control"[Mesh] OR "prevention  and  control"[sh] OR Control[tiab] OR Prevent[tiab] OR Prevention[tiab] OR Preventive[tiab]  AND  "Bedding and Linens"[Mesh] OR "Insecticides"[Mesh] OR "Permethrin"[Mesh] OR "Proguanil"[Mesh] OR "Pyrethrins"[Mesh] OR "Antimalarials"[Mesh] OR Net[tiab] OR Nets[tiab] OR Bednets[tiab] OR  Spray[tiab] OR Spraying[tiab] OR Insecticides[tiab] OR Insecticidal OR Permethrin[tiab] OR Proguanil[tiab] OR Pyrethrins[tiab] OR Pyrethroids[tiab] OR Antimalarials[tiab]  AND  Study type line  "Clinical Trials as Topic"[Mesh] OR "Epidemiologic Studies"[Mesh] OR “Morbidity"[Mesh] OR "Epidemiology"[Mesh] OR “epidemiology”[sh] OR Morbidity[tiab] OR Incidence[tiab] OR Prevalent[tiab] OR Prevalence[tiab] OR Epidemiology[tiab] |
| --- |
| **CDSR & Cochrane CENTRAL**  ([mh "Malaria"] OR [mh "Plasmodium falciparum"] OR [mh "Parasitemia"] OR Malaria:ti,ab OR Plasmodium:ti,ab OR Falciparum:ti,ab OR Parasitemia:ti,ab)  AND  ([mh "Culicidae"] OR [mh "Insect Vectors"] OR Mosquito:ti,ab OR Mosquitos:ti,ab OR Mosquitoes:ti,ab OR Vector:ti,ab OR Insect:ti,ab OR Insects:ti,ab OR Transmission:ti,ab OR  Infection:ti,ab OR Infections:ti,ab OR Infected:ti,ab)  AND  ([mh "Mosquito Control"] OR [mh /PC] OR Control:ti,ab OR Prevent:ti,ab OR Prevention:ti,ab OR Preventive:ti,ab)  AND  ([mh "Bedding and Linens"] OR [mh "Insecticides"] OR [mh "Permethrin"] OR [mh "Proguanil"] OR [mh "Pyrethrins"] OR [mh "Antimalarials"] OR Net:ti,ab OR Nets:ti,ab OR Bednets:ti,ab OR Spray:ti,ab OR Spraying:ti,ab OR Insecticides:ti,ab OR Insecticidal OR  Permethrin:ti,ab OR Proguanil:ti,ab OR Pyrethrins:ti,ab OR Pyrethroids:ti,ab OR Antimalarials:ti,ab)  AND  ([mh "Clinical Trials as Topic"] OR [mh "Epidemiologic Studies"] OR [mh "Morbidity"] OR [mh "Epidemiology"] OR [mh /EP] OR Morbidity:ti,ab OR Incidence:ti,ab OR Prevalent:ti,ab OR Prevalence:ti,ab OR Epidemiology:ti,ab) |
| **Embase**  ('Malaria'/exp OR 'Plasmodium falciparum'/exp OR 'Parasitemia'/exp OR Malaria:ti,ab OR Plasmodium:ti,ab OR Falciparum:ti,ab OR Parasitemia:ti,ab)  AND  ('mosquito'/exp OR 'disease carrier'/exp OR Mosquito:ti,ab OR Mosquitos:ti,ab OR Mosquitoes:ti,ab OR Vector:ti,ab OR Insect:ti,ab OR Insects:ti,ab OR Transmission:ti,ab OR Infection:ti,ab OR Infections:ti,ab OR Infected:ti,ab)  AND  ('insect control'/exp OR Control:ti,ab OR Prevent:ti,ab OR Prevention:ti,ab OR Preventive:ti,ab)  AND  ('bed net'/exp OR 'Insecticide'/exp OR 'Permethrin'/exp OR 'Proguanil'/exp OR 'pyrethroid'/exp OR 'antimalarial agent'/exp OR Net:ti,ab OR Nets:ti,ab OR Bednets:ti,ab OR  Spray:ti,ab OR Spraying:ti,ab OR Insecticides:ti,ab OR Insecticidal OR Permethrin:ti,ab OR Proguanil:ti,ab OR Pyrethrins:ti,ab OR Pyrethroids:ti,ab OR Antimalarials:ti,ab)  AND  ('clinical trial (topic)'/exp OR 'epidemiology'/exp OR 'Morbidity'/exp OR Morbidity:ti,ab OR Incidence:ti,ab OR Prevalent:ti,ab OR Prevalence:ti,ab OR Epidemiology:ti,ab) |
| **Web of Science (Core Collection)**  (Malaria OR Plasmodium OR Falciparum OR Parasitemia)  AND  (Mosquito OR Mosquitos OR Mosquitoes OR Vector OR Insect OR Insects OR Transmission OR Infection OR Infections OR Infected)  AND  (Control OR Prevent OR Prevention OR Preventive)  AND  (Net OR Nets OR Bednets OR Spray OR Spraying OR Insecticides OR Insecticidal OR Permethrin OR Proguanil OR Pyrethrins OR Pyrethroids OR Antimalarials)  AND  (Morbidity OR Incidence OR Prevalent OR Prevalence OR Epidemiology) |

**Table S2. Meta-analysis of different control measures against NI using the random effects model under the generalized pairwise modeling (GPM) framework in MetaXL**

| Comparison | Active | Control | RR | LCI 95% | HCI 95% |
| --- | --- | --- | --- | --- | --- |
| **UN** | UN | NI | 0.77 | 0.27 | 2.20 |
| **PD** | PD | NI | 0.16 | 0.001 | 18.48 |
| **ITN** | ITN | NI | 0.49 | 0.28 | 0.85 |
| **IRS** | IRS | NI | 0.77 | 0.34 | 1.76 |

**Overall H = 2.21**

**Table S3. Meta-analysis of different control measures against NI using the random effects model under the frequentist multivariate meta-analysis framework (mvmeta) in Stata**

| Comparison | Active | Control | RR | LCI 95% | HCI 95% |
| --- | --- | --- | --- | --- | --- |
| **UN** | UN | NI | 0.70 | 0.39 | 1.25 |
| **PD** | PD | NI | 0.15 | 0.10 | 0.22 |
| **ITN** | ITN | NI | 0.51 | 0.35 | 0.72 |
| **IRS** | IRS | NI | 0.76 | 0.39 | 1.49 |

**Table S4. Quality Scale**

| **Item** | **Questions** | **Score** |
| --- | --- | --- |
|  |  | 1=Yes/Not applicable, 0=No/Unclear |
| Design bias | | |
| 1. | What was the type of design? |  |
|  | a) randomized and allocation concealed – 2 points |  |
|  | b) randomized only – 1 points |  |
|  | c) quasi-experimental – 0 point |  |
| 2. | Was the method used to generate the sequence of randomization described and appropriate? |  |
| 3. | Was the duration of active treatment appropriate for the demonstration of study outcome? |  |
| Selection bias | | |
| 4. | Did the inclusion/exclusion criteria remain consistent across the comparison groups of the study? |  |
| 5. | Was the strategy for recruitment into the study the same across comparison groups? |  |
| 6. | Was the interval between the start of intervention and outcome the same across comparison groups? |  |
| 7. | Was attrition < 20? |  |
| Information bias | | |
| 8. | Were the outcomes of interest in the study pre-specified? |  |
| 9. | Were the outcome assessors blinded to the nature of intervention or control? |  |
| 10. | Were interventions/exposures clearly defined and implemented in the same way across both study groups? |  |
| Confounding bias | | |
| 11. | Were the groups similar at baseline in key confounding variables namely: |  |
|  | 11.1 Age-1 point |  |
|  | 11.2 Sex-1 point |  |
|  | 11.3 Owns LLINs-1 point |  |
|  | 11.4 Malaria prevelance-1 point |  |
|  | 11.5 Socio-economic status-1 point |  |
| Analytical bias | | |
| 12. | Were effect sizes based on the data available at post assessment or pre-defined subgroups rather than a post hoc portion of the data? |  |

**Table S5. Summary of the excluded studies.**

| **Study name** | **Country and year of study** | **Study population** | **Reasons for exclusion** |
| --- | --- | --- | --- |
| Alonso, 1993 | The Gambia, 1988 | Children: 0-5 years | No comparison group |
| Bojang, 2010 | The Gambia, 2007-08 | Children: 6-59 months | No comparison group |
| Bradley, 1986 | The Gambia, 1981 | Children <7 years | Cross-sectional survey |
| Bradley, 2013 | Equatorial Guinea | Children:2-14 years | Survey |
| Carnevale, 1991 | The Gambia, Burkina Faso and Tanzania | All ages | Failure to access full article |
| Chandramohan, 2005 | Ghana, 2000 | Children: <1 year | Single dose (>1 months) |
| Cisse, 2009 | Senegal, 2007 | Children: <5 year | No comparison group |
| Dicko, 2008 | Mali, 2002 | Children: 6/12-10 years | Single dose (>1 months) |
| Gimnig, 2016 | Kenya, 2008-09 | All ages | Cross-sectional survey |
| Gosling, 2009 | Tanzania, 2004-08 | Infants: 8-12 weeks | Single dose (>1 months) |
| Graves, 1987 | Papua New Guinea, 1985 | Children: <10 years | Cross-sectional survey |
| Graves, 2008 | Eritria, 1998-2003 | All ages | Case control studies |
| Guyatt, 2002 | Kenya, 2000 | All ages | No follow up time |
| Habluezel, 1999 | Burkina Faso, 1994 | Children: 1/2-59 months | Insecticide-treated Curtains |
| Halliday, 2014 | Kenya, 2010-12 | School children: 5-20 years | Screening |
| Hamusse, 2012 | Ethiopia, 2001-02 | All ages | Cross-sectional survey |
| Hii, 1987 | Malausia, 1984-85 | All ages | Cross-sectional survey |
| Kimani, 2006 | Kenya, 2002 | All ages | Pre-post intervention |
| Kleinschmidt, 2006 | Equatorial Guinea, 2003 | Children: 2-14 years | Pre-post intervention |
| Kleinschmidt, 2009 | Equatorial Guinea, Zambia and Mozambique, 2006-08 | Children: 2-14 years | Cross-sectional survey |
| Klinkenberg, 2010 | Ghana, 2004 | Children: 0-72 months | Cross-sectional survey |
| Kobbe, 2007 | Ghana, 2003-05 | Infants | Single dose (>1 months) |
| Kobbe, 2007 | Ghana, 2003-06 | Infants | Spatio-temporal study |
| Kweku, 2009 | Ghana, 2006 | Children: 3-59 months | Delivery method study |
| Leestra, 2003 | Kenya, 1999 | Children: 12−18 years | Cross-sectional survey |
| Liljander, 2010 | Ghana, 2005-06 | Children: 3-59 months | Cross-sectional survey |
| Lindblade, 2015 | Malawi, 2011-12 | Children: 6-59 months | No comparison group |
| Louis, 2015 | Burkina Faso, 2009 & 2011 | Children: 2 weeks to 36 months | Pre-post intervention |
| Lyimo, 1991 | Tanzania, 1987 | Children: 1-10 months | No comparison group |
| Macete, 2006 | Mozambican, 2002 &2004 | Infants | Single dose (>1 months) |
| Magris, 2007 | Venezuela, 1998 | All ages | Curtains, Hammocks, bed sheets |
| Mashauri, 2013 | Tanzania, 2007-08 | All ages | Cross-sectional survey |
| Menendez, 2008 | Mozambique, 2003 & 2005 | Pregnant women | Placental infection |
| Mockenhaupt, 2007 | Ghana, 2003 & 2005 | Infants | Single dose (>1 months) |
| Nevill, 1996 | Kenya, 1991-93 | Children: <5 years | Severe malaria illness |
| Pardo, 2006 | Equatorial Guinea, 2004-05 | Children: 0-5 years | Cross-sectional survey |
| Phiri, 2012 | Malawi, 2006-09 | Children: 4-59 months | Severe malaria illness |
| Premji, 1995 | Tanzania, 1992-93 | Children: 6-40 months | Cross-sectional survey |
| Procacci, 1991 | Burkina Faso, 1987-89 | Children: 6 months-6 years | Curtains, Hammocks, bed sheets |
| Rowland, 1994 | Afghanistan, 1995 | Children: 5-15 years | Pre-post intervention |
| Rowland, 1999 | Afghanistan, 1996 | All ages | Curtains, Hammocks, bed sheets |
| Rowland, 2000 | Pakistan, 1997 | All ages | Pre-post intervention |
| Rowland, 2002 | Afghanistan, 1995-96 | All ages | Pre-post intervention |
| Sarpong, 2015 | Ghana, 2012 | Children: 2-14 years | Cross-sectional survey |
| Senn, 2012 | Papua New Guinea, 2006-10 | Infants | Single dose (>1 months) |
| Shanks, 1998 | Kenya, 1996 | Adults: 18-65 years | Efficacy and safety studies |
| Sharp, 2002 | Zambia, 2000 | Adults | Pre-post intervention |
| Sharp, 2007 | Mozambique, South Africa & Swaziland, 2000-04 | All ages | No comparison group |
| Sievers, 2008 | Rwanda, 2006 | Children: 0-12 years | Pre-post intervention |
| Skarbinski, 2011 | Malawi, 2009 | Children: <5 years | No comparison group |
| Skarbinski, 2012 | Malawi, 2007 | Children: <5 years | No follow up time |
| Sokhna, 2008 | Senegal, 2004 | Children: 3-59 months | No comparison group |
| Sutanto, 1999 | Indonesia, 1993-95 | All ages | Cross-sectional survey |
| ter Kuile, 2003 | Kenya, 1996-99 | Children: <5 years | Cross-sectional survey |
| Thang, 2009 | Vietnam, 2004 | All ages | Curtains, Hammocks, bed sheets |
| Tseng, 2008 | São Tomé and Príncipe, 2004 | Children: <9 years | Pre-post intervention |
| West,2014 | Tanzania, 2012 | Children: 6 months- 14 years | Cross-sectional survey |
| Zhou, 2010 | Kenya, 2005 | Children: 1-5 years | No denominator |

**Table S6. Drugs used in the included studies**

| 1. Proguanil hydrochloride (100 mg) was given based on the weight of the participants (one tablet for those ≤ 30 kg and two tablets if the weight was 31-60 kg);^45^ |
| --- |
| 1. One dose of SP- sulfadoxine 25mg/kg + pyrimethamine 1.25mg/kg (Cosmos, Nairobi, Kenya) and one dose of artesunate 4mg/kg (Sanofi Synthelabo, Paris, France) once a month for 3 months.^35^ |
| 1. SP was given at a dose of 175/8·75 mg to children 5–9 kg, 350/17·5 mg to children 10–18 kg, and 550/26.25 mg to those who weighed 19 kg. The corresponding doses for AQ were 70 mg, 140 mg, and 220 mg. AQ was given over 3 days.^36^ |
| 1. Mefloquine (salt; Lariam [US formulation]; Hoffmann–La Roche) 250 mg/week for 12 weeks.^38^ |
| 1. SP was 25/1.25 mg per kg, and AQ was 10 mg per kg daily for 3 days. The first dose of AS was given with SP.^40^ |
| 1. Children: 3-5 months received ¼ tablet; 6-11 months ½ tablet; 12-23 months ¾ tablet; ≥24 months one tablet each of SP, co-formulated AS and AQ was given every 28 days on six ocassions; ^41^ |
| 1. Dihydroartemisinin-peperaquine (DP) (Duo-Cotecxin, Holley-Cotec Pharmaceuticals, Beijing, China) containing 120 mg of dihydroartemisinin (DHQ) and 960 of peperaquine (PQ) was given once daily for 3 consecutive days each month; ^57^ |
| 1. DP (Duo-Cotecxin, Holley-Cotec Pharmaceuticals, Beijing, China) was given with dosage of 6.4 mg/kg dihydroartemisinin abd 51.2 mg/kg peperaquine once daily for 3 consecutive days each month;^44^ |
| 1. SP-AS3 (DAFRA Pharma, Belgium): 250 mg sulphadoxine, 12.5 mg pyrimethamine once daily of treatment (followed by a placebo SP tablets on days 2 and 3) and 25 mg AS once daily for 3 days;^46^ |
| 1. Either SP or AQ (Kina Pharma Ltd, Accra, Ghana) were given daily for three days to the participants, Children aged 3-11 months received one quarter of tablet, 1-2 years of age received half while 3-5 years old was given a whole tablet. ^48^ |
| 1. Azithromycin 750 mg loading dose followed by 250 mg/days was given to participants for 20 weeks;^54^ |

**Table S7. Description of ITN’s used across the studies**

| 1. Nets were also impregnated with permethrin (Peripel 10^®^, Welcome Foundation, UK) at concentration of 0.5 g/m^2^;^33,49,52^ |
| --- |
| 1. Olyset nets was one type of ITN (LLIN) which had factory treated with permethrin were made of blue polythene of 150 denier ster with 72 mesh (8X9 holes/in2);^56,58^ |
| 1. Similarly nets were treated with permethrin (0.5 g/m^2^);^37^ |
| 1. LLINs were treated with deltamethrin (TianJin-Yorkool Ltd, Tianjian, PR China, and Lantrade Global Supplies Ltd, Gerrards Cross, UK).^60^ |
| 1. Bed nets were impregnated with permethrin in Muisne, Borbon and Madre de Dios and with lambdacyhalothrin in San Juan and Catacaos.^61^ |
| 1. ITN (Siamdutch Mosquito Netting Co., Bangkok, Thailand) pre-impregnated with the target dost of 0.5g of permethrin/m^2^ were used.^53^ |

**Table S8. Description of IRS treatments used across the included studies**

| 1. The interior walls of all turkels in the intervention camps were sprayed with malathion at 2 g/m^2^ over a 5-day period.^34^ |
| --- |
| 1. Lambda-cyhalothrin capsule suspension (ICON CS; Syngenta, AG, Midrand, South Africa).^39^ |
| 1. Lambda-cyhalothrin (0.05%);^108^ |
| 1. DDT (DDT 75% wettable powder; Hindustan Insecticides, New Delhi, India) at a target dose of 2 g/m² to dwelling rooms.^47^ |

**Table S9. Quality assessment scores of included studies**

| **Study** | **1** | **2** | **3** | **4** | **5** | **6** | **7** | **8** | **9** | **10** | **11.1** | **11.2** | **11.3** | **11.4** | **11.5** | **12** | **Score** | **Study design** |
| --- | --- | --- | --- | --- | --- | --- | --- | --- | --- | --- | --- | --- | --- | --- | --- | --- | --- | --- |
| Cisse, 2006 | 2 | 1 | 1 | 1 | 1 | 1 | 1 | 1 | 1 | 1 | 1 | 1 | 0 | 1 | 0 | 1 | 15 | PD -NI |
| Dicko, 2011 | 2 | 1 | 1 | 1 | 1 | 1 | 1 | 1 | 1 | 1 | 1 | 1 | 0 | 0 | 0 | 1 | 14 | PD -NI |
| Hale, 2003 | 2 | 1 | 1 | 1 | 1 | 1 | 1 | 1 | 1 | 1 | 1 | 1 | 0 | 0 | 0 | 1 | 14 | PD -NI |
| Konate, 2011 | 2 | 1 | 1 | 1 | 1 | 1 | 1 | 1 | 1 | 1 | 1 | 1 | 1 | 0 | 0 | 1 | 15 | PD -NI |
| Kweku 2008 | 2 | 1 | 1 | 1 | 1 | 1 | 1 | 1 | 1 | 1 | 0 | 1 | 1 | 0 | 0 | 1 | 14 | PD -NI |
| Lwin, 2012 | 2 | 1 | 1 | 0 | 0 | 1 | 0 | 1 | 1 | 1 | 0 | 1 | 0 | 0 | 0 | 1 | 10 | PD -NI |
| Nankabirwa, 2014 | 2 | 1 | 1 | 1 | 1 | 1 | 1 | 1 | 1 | 1 | 1 | 1 | 1 | 1 | 1 | 1 | 17 | PD -NI |
| Odhiambo, 2010 | 2 | 1 | 1 | 1 | 1 | 1 | 1 | 1 | 1 | 1 | 1 | 1 | 1 | 0 | 0 | 1 | 15 | PD -NI |
| Sesay, 2011 | 2 | 0 | 1 | 1 | 1 | 1 | 1 | 0 | 1 | 1 | 1 | 1 | 1 | 1 | 0 | 1 | 14 | PD -NI |
| Taylor, 1998 | 2 | 1 | 1 | 1 | 1 | 1 | 0 | 1 | 1 | 1 | 1 | 1 | 0 | 1 | 0 | 1 | 14 | PD -NI |
| Charlwood, 2001 | 1 | 0 | 1 | 1 | 1 | 1 | 1 | 1 | 0 | 1 | 0 | 0 | 0 | 0 | 0 | 1 | 9 | IRS-NI |
| Hamel, 2011 | 0 | 0 | 1 | 1 | 1 | 1 | 1 | 1 | 0 | 1 | 1 | 1 | 0 | 1 | 0 | 1 | 11 | IRS-NI |
| Pinder, 2015 | 1 | 1 | 1 | 1 | 1 | 1 | 1 | 1 | 1 | 1 | 1 | 1 | 1 | 1 | 1 | 1 | 16 | IRS-NI |
| Kamol-Ratanakul, 1992 | 2 | 1 | 1 | 1 | 1 | 1 | 1 | 1 | 1 | 1 | 1 | 1 | 1 | 0 | 0 | 1 | 15 | ITN-UN |
| Luxemburger, 1994 | 2 | 1 | 1 | 1 | 1 | 1 | 1 | 1 | 1 | 1 | 1 | 1 | 1 | 1 | 0 | 1 | 16 | ITN-UN |
| Sharma, 2009 | 1 | 1 | 1 | 1 | 1 | 1 | 1 | 1 | 0 | 1 | 1 | 1 | 0 | 0 | 0 | 1 | 12 | ITN-UN |
| Soleimani-Ahmad, 2012 | 1 | 1 | 1 | 1 | 1 | 1 | 1 | 1 | 0 | 1 | 0 | 0 | 0 | 0 | 0 | 1 | 10 | ITN-UN |
| Beach, 1993 | 0 | 0 | 1 | 1 | 1 | 1 | 0 | 1 | 0 | 1 | 1 | 1 | 0 | 0 | 0 | 1 | 9 | ITN-NI |
| Fraser-Hurt, 1999 | 1 | 1 | 1 | 1 | 1 | 1 | 1 | 1 | 0 | 1 | 1 | 0 | 0 | 0 | 0 | 1 | 11 | ITN-NI |
| Hill, 2014 | 1 | 1 | 1 | 1 | 1 | 1 | 1 | 1 | 1 | 1 | 1 | 1 | 0 | 0 | 0 | 1 | 13 | ITN-NI |
| Kroeger, 1995 | 1 | 1 | 1 | 0 | 0 | 1 | 1 | 1 | 0 | 1 | 0 | 0 | 0 | 0 | 0 | 1 | 8 | ITN-NI |
| Marbiah, 1998 | 1 | 0 | 1 | 1 | 1 | 1 | 1 | 1 | 1 | 1 | 0 | 0 | 0 | 0 | 0 | 1 | 10 | ITN-NI |
| Rowland, 1996 | 1 | 1 | 1 | 1 | 1 | 1 | 1 | 1 | 1 | 1 | 1 | 1 | 0 | 1 | 0 | 1 | 14 | ITN-NI |
| Sahu, 2003 | 1 | 1 | 1 | 1 | 1 | 1 | 0 | 1 | 0 | 0 | 0 | 0 | 0 | 0 | 0 | 1 | 8 | ITN-NI |
| Sexton, 1990 | 1 | 1 | 1 | 1 | 1 | 1 | 1 | 1 | 0 | 1 | 1 | 1 | 0 | 0 | 0 | 1 | 12 | ITN-NI |
| Shah, 2013 | 0 | 0 | 1 | 1 | 1 | 1 | 0 | 1 | 0 | 1 | 0 | 0 | 0 | 0 | 0 | 1 | 7 | ITN-NI |
| Smithuis, 2013 | 1 | 1 | 1 | 1 | 1 | 1 | 1 | 1 | 0 | 1 | 1 | 1 | 0 | 0 | 0 | 1 | 12 | ITN-NI |
| Nevill, 1988 | 0 | 0 | 1 | 1 | 1 | 1 | 0 | 1 | 0 | 1 | 1 | 1 | 0 | 0 | 0 | 1 | 9 | UN-PD |
| Mwangi, 2003 | 0 | 0 | 1 | 0 | 0 | 1 | 1 | 1 | 0 | 1 | 1 | 1 | 0 | 0 | 0 | 1 | 8 | UN-NI |
| Snow, 1988 | 1 | 1 | 1 | 1 | 1 | 1 | 0 | 1 | 0 | 1 | 1 | 1 | 0 | 0 | 0 | 1 | 11 | UN-NI |
| Percentage of studies missing the safe-guards | 16.7 | 26.7 | 0 | 10.0 | 10.0 | 0 | 23.3 | 3.3 | 46.7 | 3.3 | 26.7 | 23.3 | 73.3 | 73.3 | 93.3 | 0 |  |  |
