# Supplementary figures and images for: Comparative effectiveness of malaria prevention measures: a systematic review and network meta-analysis
Source: Parasit Vectors. 2018 Mar 27;11:210. doi: 10.1186/s13071-018-2783-y (PMC5869791; doi:10.1186/s13071-018-2783-y)

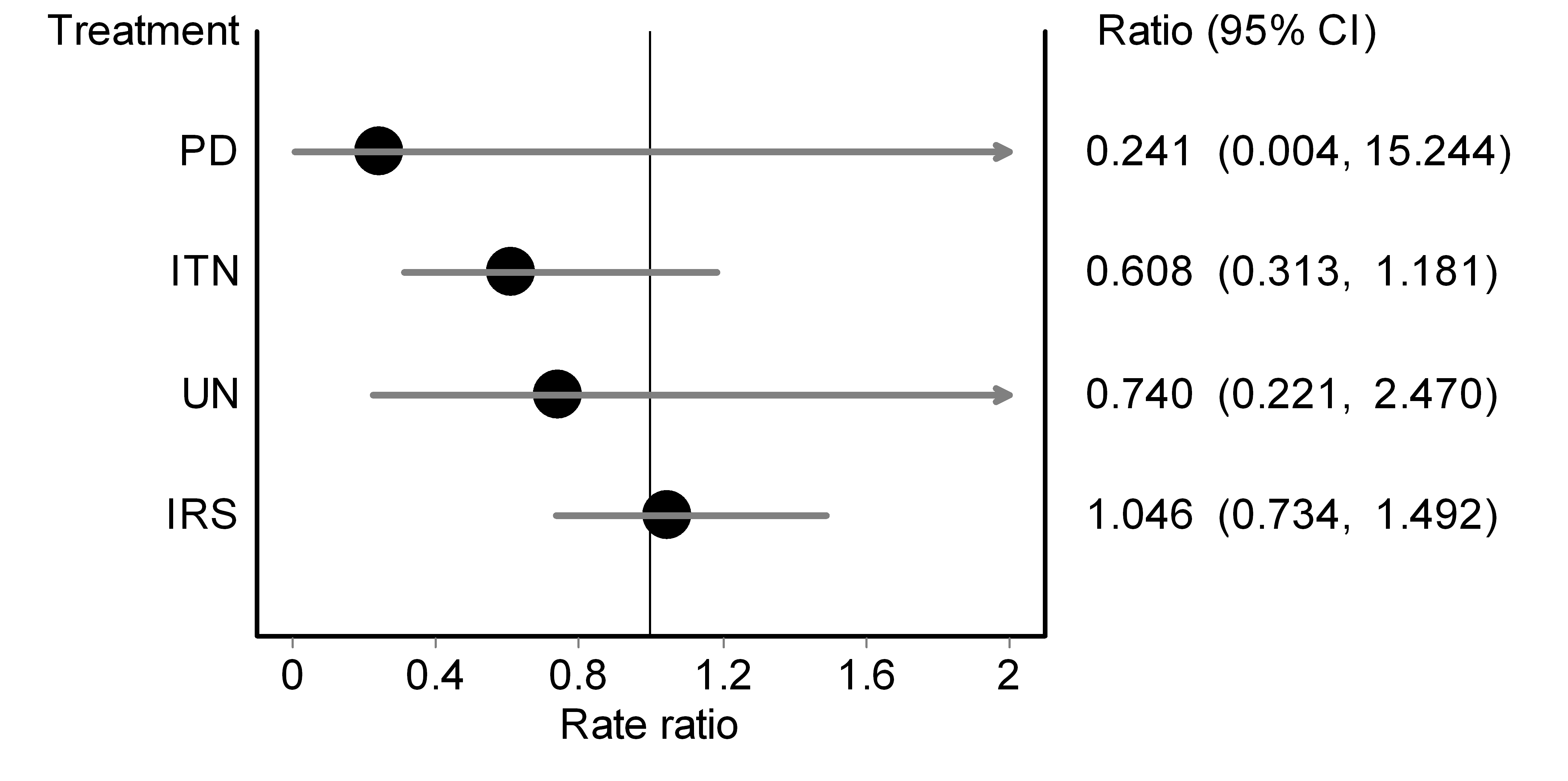

Supplement: Supplementary file 2 — Figure S1. Results of network meta-analysis of 21 studies with children as a study population. (TIFF 624 kb) [file 13071_2018_2783_MOESM2_ESM.tif]

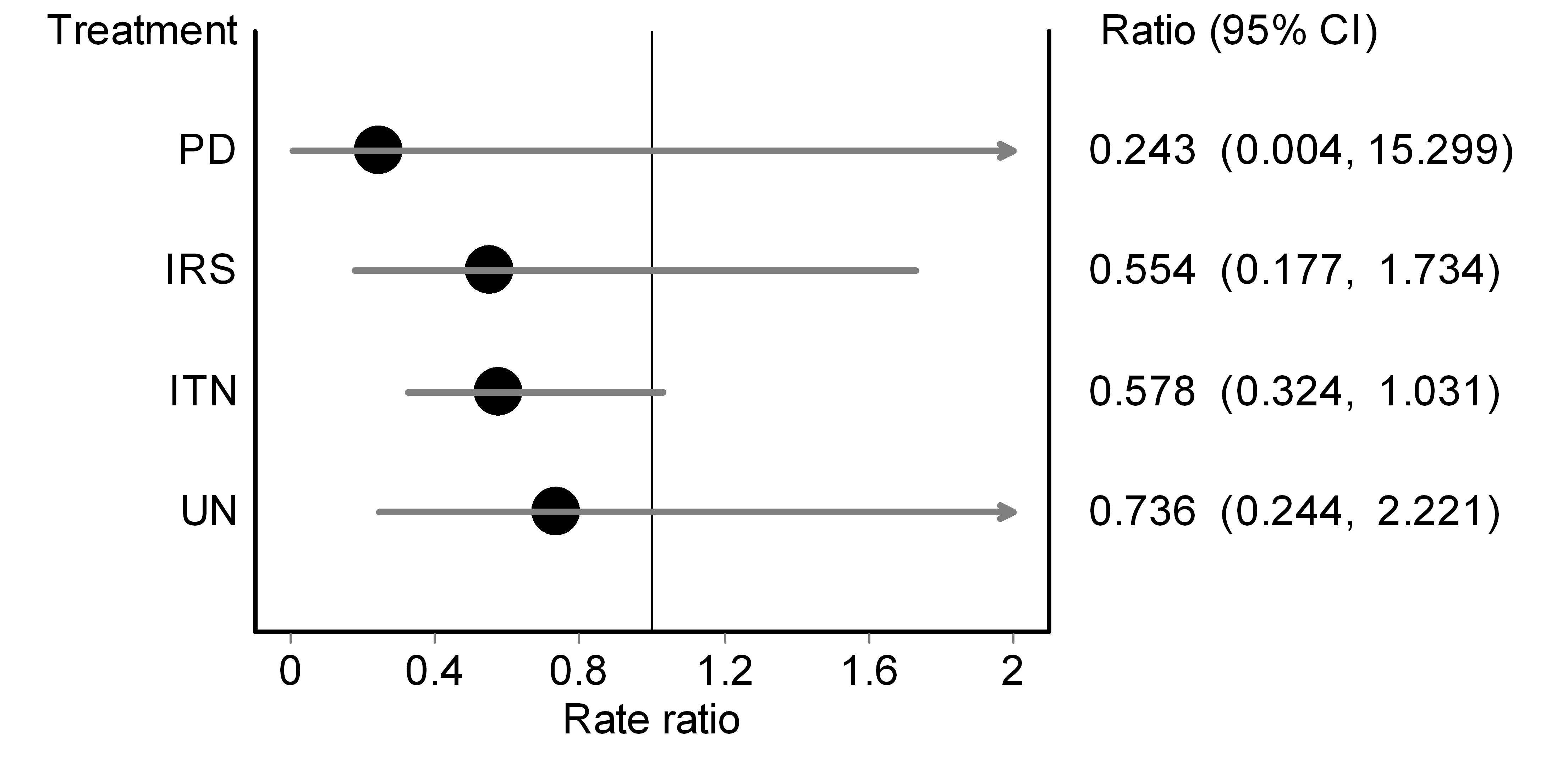

Supplement: Supplementary file 3 — Figure S2. Results of network meta-analysis of 28 studies with incidence of Plasmodium falciparum. (TIFF 631 kb) [file 13071_2018_2783_MOESM3_ESM.tif]

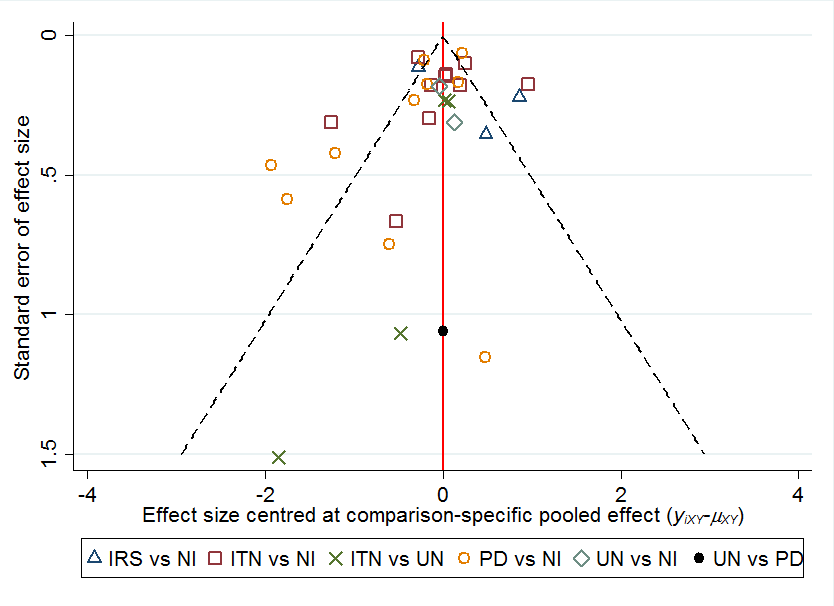

Supplement: Supplementary file 4 — Figure S3. Funnel plot depicting asymmetry for the PD-NI comparison. (TIFF 1482 kb) [file 13071_2018_2783_MOESM4_ESM.tif]
